# Combined deep and reinforcement learning with gaming to promote healthcare in neurodevelopmental disorders: a new hypothesis

**DOI:** 10.3389/fnhum.2025.1557826

**Published:** 2025-03-14

**Authors:** Fabrizio Stasolla, Anna Passaro, Enza Curcio, Mariacarla Di Gioia, Antonio Zullo, Mirella Dragone, Elvira Martini

**Affiliations:** ^1^University Giustino Fortunato of Benevento, Benevento, Italy; ^2^Universitas Mercatorum of Rome, Rome, Italy

**Keywords:** neurodevelopmental disorders, gaming, artificial intelligence, deep learning, reinforcement learning, quality of life, healthcare

## Introduction

Children and adolescents with neurodevelopmental disorders (NDDs) may experience significant problems dealing with daily activities and/or everyday life environmental requests. Besides intellectual disabilities, communication disorders and challenging behaviors may occur. Commonly, isolation and passivity are acknowledged. Accordingly, social interactions may be relevantly compromised. NDD usually has an early onset, a variable clinical manifestation, a wide range of severity, and a recognized comorbidity (Howner et al., 2018; Malik et al., 2023). Their clinical conditions may have negative outcomes on their quality-of-life, and families' and caregivers' burden may be meaningfully increased with negative consequences for their overall management and healthcare (Kanniappan et al., 2024; Lefton-Greif et al., 2024; Materula et al., 2023). An early assessment is crucial to tackle this issue (Chorna et al., 2024; Henry et al., 2016), for which standard tests or technology-based solutions are available (Ceruti et al., 2024; Niu et al., 2022; Woodcock and Blackwell, 2020). Traditional assessment relies on neuropsychological evaluation (Haddad et al., 2024; Hamadelseed et al., 2023). Technology-aided options may represent a functional bridge between personal skills and environmental requests by enhancing self-determination and positive occupation accordingly (Passaro et al., 2024). Recently, artificial intelligence (AI)-based programs emerged (Boubakri and Nafil, 2024). Both assessment and rehabilitative goals are targeted (Anbarasi et al., 2024; Climent-Pérez et al., 2024).

Deep learning (DL), as part of machine learning (ML) solutions, has been growingly used to evaluate normal brain functioning and differentiate between individuals who have normal development and individuals who are at risk of developmental disorders (Kucewicz et al., 2023; Li et al., 2024; Swinckels et al., 2024). For example, DL algorithms as convolutional neural networks (CNNs) have progressed, allowing future learning of a significant amount of data patterns, enabling subjectivity in future extraction procedures. Successful implementations of DL and CNN have been documented. Thus, brain functioning has been positively investigated through functional magnetic resonance imaging (fMRI) in the main domains (Hu et al., 2023).

Reinforcement learning (RL), as a further part of ML, has been adopted for rehabilitative purposes. That is, an artificial intelligence agent continuously interacting with a participant dealing with a cognitive task is positively reinforced by such interaction and can learn from it. Based on the interaction, it will provide the participant with an optimal task. Consequently, the artificial intelligence agent ensures the participants with highly customized and tailored solutions during all working sessions, and an ideal learning process will be ensured (Zini et al., 2022). Recently, RL-based principles have been adopted for emotional regulation in neurodevelopmental disorders and neurodegenerative diseases (Stasolla et al., 2024; Stasolla and Di Gioia, 2023).

Gamification may be considered an advanced technological cornerstone in neurodevelopmental disorders for both assessment and rehabilitative purposes. Educational and recovery goals may be targeted. Education, healthcare, and rehabilitation objectives have been pursued. Significant improvements have been reported in individuals with developmental disabilities. Self-determination, independence, and fulfillment may be fostered by embedding features such as challenges, competitions, and rewards. Thus, gamification can help persons with neurodevelopmental disorders by enhancing active roles and constructive engagement (Boubakri and Nafil, 2024).

A literature overview was performed on Scopus. Neurodevelopmental disorders, quality of life, DL, RL, gamification, assessment, and rehabilitation were merged as keywords. Although detailed solutions were widely adopted in neurodevelopmental disorders (Alves et al., 2020; Bakir et al., 2023; Brzosko et al., 2019; Nahar et al., 2024; Ouyang et al., 2024; Pandya et al., 2024; Rahman et al., 2024; Rodulfo-Cárdenas et al., 2023; Wyatt et al., 2024; Zhao et al., 2024), no records were found on their integration. In line with the above, the aims of the current opinion paper were (a) to provide the reader with a concise framework on the use of DL, RL, and gaming in developmental disorders, (b) to propose a new hypothesis on their combined use to evaluate between normal development and individuals who may be at risk of neurodevelopmental disorders, (c) to critically argue on their matching for assessment and rehabilitative objectives, and (d) to discuss the implications for research and clinical practice. Limitations and future research perspectives were additionally highlighted.

## DL and neurodevelopmental disorders

DL in neurodevelopmental disorders is a widely adopted strategy of assessment with 63 records found, including five reviews published between 2020 and 2024 (Kucewicz et al., 2023; Li et al., 2024; Soybilgic and Avcin, 2020; Swinckels et al., 2024; Wang et al., 2023). Li et al. (2024) conducted a comprehensive review of the use of electroencephalography (EEG) as a method that records changes in brain activity, which may represent a marker for the identification of autism spectrum disorders (ASDs). The review included DL and ML methods. Future perspectives and challenges were highlighted to automatically diagnose ASDs through EEG signals to emphasize ASD automated identification. Kucewicz et al. (2023) summarized the outcomes of different invasive approaches to brain stimulation to modulate memory functions. The challenges faced in the initial investigation of memory were evidenced. A classification of the various stimulation approaches into continuous and phasic modulation with an open- or closed-loop responsive stimulation based on the analysis of neural activities was detailed. Implantable devices for high-density recording, stimulation of EEG activities, and technologies for distributed brain–computer interface emerged as future avenues for research and clinical practice. Soybilgic and Avcin (2020) reviewed recent findings on antiphospholipid pediatric syndrome (APS) in children and neonates. New diagnostic criteria for an accurate diagnosis of APS were investigated. A regular assessment of neurodevelopmental status was warranted. DL may represent a valid strategy for early assessment.

Swinckels et al. (2024) carried out a scoping review of evidence on how the use of ML in electronic health records (EHRs) could help support the early detection of disease. Medical insights and clinical benefits were considered by reviewing applications used in different diseases. NDDs were additionally examined. ML were combined with EHR models. Specifically, DL emerged as a valid strategy of detection or prediction if compared to standard clinical assessment. Although ML models based on textual EHRs are in the developmental stage of advancement, they are viewed as a critical and invaluable strategy for supporting clinicians and researchers in an early diagnosis. Wang et al. (2023) analyzed multimodal magnetic resonance imaging (MRI) data from the existing literature and reviewed the abnormal changes in brain structural and functional networks among children with ASDs. Structural MRI emphasized morphological differences, abnormal developmental trajectories, and network connectivity changes in the brain at different ages. Functional MRI emphasized disruption of functional networks, abnormal perfusion, and neurovascular decoupling associated with ASD symptoms. MRI multimodal was recognized as a valid strategy to detect early diagnosis of ASDs through DL principles.

## RL and neurodevelopmental disorders

RL in neurodevelopmental disorders is also represented in the literature, with 60 documents available in Scopus and five reviews in the last two decades (Brzosko et al., 2019; Meyer et al., 2005; Rodulfo-Cárdenas et al., 2023; Swan et al., 2016; Wyatt et al., 2024). By further inspection, Meyer et al. (2005) investigated an animal model for the early detection of schizophrenia, which was considered irrelevant to the current study and was not detailed accordingly. Brzosko et al. (2019) reviewed the neuromodulation of spike-timing-dependent plasticity (STDP), viewed as a leading cellular model for behavioral learning and memory with rich computational properties. Neuromodulation provides an attractive way to connect these different timescales, and strong experimental evidence exists on the connection between STDP and neuro-modulatory control by acetylcholine, monoamines, and other signaling molecules. The modulation of STDP was critically reviewed, functional implications were highlighted, and useful insights for future research were argued. Rodulfo-Cárdenas et al. (2023) systematically reviewed the literature on the relationship between early exposure to particulate matter (PM) as a major component of ambient pollution and neurodevelopmental outcomes in experimental studies. Eleven studies with postnatal exposure and nine studies with both pre and postnatal exposure were included. Data suggested that exposure to PM can negatively determine human development, triggering disorders in short-term memory, sociability, and impulsivity. Alterations in synaptic plasticity were additionally documented in the immune system. Differences between the sexes emerged. A time-sex interaction occurred. The postnatal period was more important, and male patients were more affected. Further experimental investigations were warranted to prioritize examining learning impulsivity and biochemical parameters with a specific interest in differences between sexes.

Swan et al. (2016) targeted the generalization process of therapeutic gains across settings, stimuli, and time. Research outcomes supported the use of positive reinforcement for adaptive behaviors and altered the maladaptive contingencies with challenging behaviors, which were associated with positive results. Different training conducted by applying therapy skills across contexts and systematically varying stimuli also had beneficial effects and positive clinical implications for internalizing and externalizing behaviors among neurodevelopmental disorders. The use of technology was specifically sought. Wyatt et al. (2024) conducted a systematic review of fMRI, exploring and exploiting the decision-making process in healthy adults during foraging, RL, and information research. Exploration decision-making was associated with the engagement of attentional, control, and salience networks. Conversely, exploitation decision-making was associated with default network brain regions. Data were interpreted in the context of a network architecture that is useful to support flexible switching between externally and internally directed cognitive processes, which is mandatory for adaptive and purposeful behaviors. Moreover, they surveyed studies involving neurodevelopmental, neuropsychological, and neuropsychiatric disorders, as well as lifespan development and neurodegenerative diseases. Significant differences between exploring and exploiting decision-making were observed across the populations, corroborating that the two decision-making modes are supported by independent neural circuits. Comprehensive neural circuit mapping and neural behavioral correlates associated with exploration-exploitation in humans were warranted. A new trans-diagnostic approach to assessment, surveillance, and intervention for cognitive decline and dysfunction in mental health for normal development and the clinical population was put forward.

## Gamification and neurodevelopmental disorders

Gamification and neurodevelopmental disorders are represented in Scopus with three reviews (Alves et al., 2020; Bakir et al., 2023; Boubakri and Nafil, 2024). Alves et al. (2020) combined applied behavior analysis-based interventions (ABAs) and technology-aided programs to help individuals with ASDs. Gamification, software apps, computer-based training (Web), and robotics were detailed. The features of these technologies were specified. The reviewed protocols focused on technologies such as distributed systems, image processing, gamification, and robotics. The primary goals of the tools and devices were aimed at enhancing communication, social interactions, and reading skills. Bakir et al. (2023) systematically reviewed the use of augmented reality (AR) in mental-health clinical conditions. Four main categories of studies were identified, namely (a) neurodevelopmental disorders, (b) anxiety and phobia, (c) psychoeducation and wellbeing, and (d) procedural and pain management. Results demonstrated the effectiveness of AR-based interventions in mental health-related conditions. However, a high heterogeneity and a small sample recommended further research addressing a larger sample and high-quality study designs.

Boubakri and Nafil (2024) explored the potential impact of gamification on accessibility issues for persons with disabilities. According to PRISMA guidelines, a literature review was performed across seven databases. Fifty-three studies were selected. Data revealed that gamification was suitable for neurodevelopmental disorders, blindness, and visual impairments, relevantly improving the learning and rehabilitation processes of individuals with disabilities. Nevertheless, significant gaps remain to be filled, including the need for a comprehensive assessment framework and a more accurate integration with emerging technologies such as AI-based programs to customize optimal solutions for individuals with disabilities. Although gamification was proven to be effective and suitable for targeting challenges in persons with disabilities, further balanced perspectives were required. Specifically, a focused approach to gamification to identify its potential benefits as well as to address the existing gaps and claims for its integration with emerging technologies is used to generate more inclusive and tailored experiences.

An illustrative example might be represented by a combined immersive system, including AR, VR, and gamification. One can argue that a highly immersive and funny system may promote executive functions, communicative skills, and social interactions. Different rigorously customized solutions may be designed. Based on RL principles, one may envisage different tasks properly tailored to the participant's capacities and continuously adapted to his/her performance. Both cognitive skills and behavioral responses may be recorded, monitored, and tracked. Recently, Stasolla et al. (2025) proposed a suitable scoping review on this specific topic.

## The combined solution: a new hypothesis

**Figure 1:**
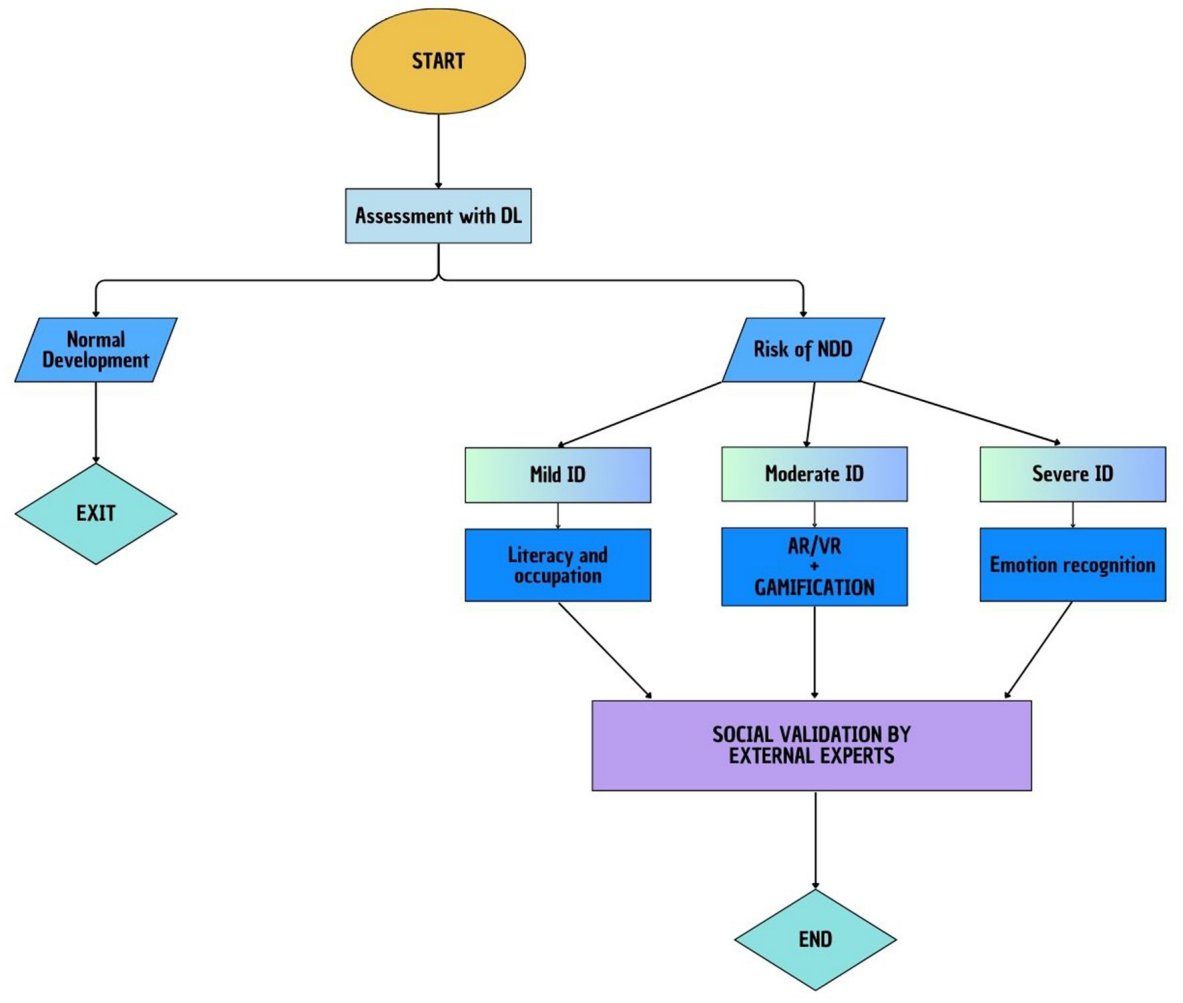
Three-step hierarchical proposal.

Considering the above, a new hypothesis on a combined and integrated technological solution based on DL, RL, and gamification principles was proposed. Technological options have been recently outlined (Stasolla et al., 2023). A three-step hierarchical solution was recommended for individuals with NDDs for assessment and rehabilitative purposes (see Figure 1). One may design a first step in which, through DL principles, the artificial system will be capable of differentiating between individuals who may have normal development and individuals who are at risk of NDDs through exploring and mapping the brain activity during cognitive tasks. Once differentiated, with a combined solution of RL and gamification (i.e., second step), one may plan funny, educational, and rehabilitative tasks focused on promoting cognitive and communicative skills as well as redirecting challenging behaviors into adaptive, functional, or occupational activities (Chiapparino et al., 2011). In a third step, the clinical validity may be assessed by involving expert external raters in social validation procedures (Stasolla et al., 2019). Different solutions may be adapted and customized depending on the participant's functioning.

For instance, for individuals with severe to profound intellectual and multiple disabilities, one may be supposed to design a basic discrimination between positive and negative emotions or situations eliciting specific emotions. For individuals with a moderate level of intellectual disabilities, one may plan combined AR, virtual reality (VR), and gamification opportunities to enhance different social interactions. For individuals with high intellectual functioning and ASDs or for persons estimated as borderline between the normal range of intellectual functioning and mild intellectual disabilities, one may implement access to literacy integrated with occupational and/or leisure opportunities (Chiapparino et al., 2011; Lancioni et al., 2007). Finally, one may envisage combined rehabilitative programs focused on supporting academic skills, personal needs, and access to literacy (Stasolla et al., 2022). Different technological solutions may be considered to provide a fully immersive environment (Bennewith et al., 2024; Panzeri et al., 2024).

## Discussion

Individuals with NDDs and different levels of disabilities may experience significant difficulties in different daily contexts and settings. Because they commonly present intellectual, motor, communicative, and sensorial impairments, they constantly rely on families' and caregivers' assistance. This condition may be deleterious for their social image, status, and desirability. It may seriously hamper their quality of life. To overcome this issue, technology-aided solutions may be helpful, as previous findings have demonstrated (Kinsella et al., 2017; Paul et al., 2023; Pham et al., 2019). Recently, AI-based solutions emerged as a valid avenue to promote healthcare for children and adolescents with NDDs (Mengi and Malhotra, 2022). Here, a new hypothesis about a combined technological solution was proposed. The following considerations were suggested.

First, self-determination, constructive engagement, independence, and social inclusion were fostered. Thus, an active role, positive occupation, and functional activities were supported by reduced isolation and passivity. Assessment and rehabilitation were critically targeted issues (Harris, 2020; Kwan et al., 2021; Lancioni et al., 2008, 2004; Stasolla et al., 2014b,a; Zimmer and Dunn, 2021).

Second, different behaviors depending on the individuals' levels of functioning may be outlined. For example, one may envisage a simple discrimination between positive and negative emotions for individuals with very low functioning and limited behavioral repertoire. Otherwise, one may design to promote communication and positive social interactions for individuals with a moderate level of disabilities. Furthermore, more complex programs focused on different hierarchical managed opportunities may be considered (Michalski et al., 2021).

Third, one may include an assessment of brain activity and a rehabilitative intervention in a unique program. Thus, both evaluation and recovery issues may be embedded. Through DL systems, one may differentiate between individuals with normal development and those at risk of neurodevelopmental disorders. Gamification may play a dual crucial role in educational and rehabilitative purposes (Gao et al., 2024; Liu et al., 2024; Shariat et al., 2024).

Fourth, families' and caregivers' burden may be reduced. The clinical validity of the proposed intervention may be assessed. One may argue that by being constructively engaged and positively occupied with highly customized and tailored solutions, individuals with NDDs may be more easily involved and included in daily settings with a burden reduction, and a more effective and suitable solution was adopted (Song et al., 2022).

Despite the promising postulated outcomes, some relevant issues should be acknowledged. First, no empirical data were available. Systematic reviews, metanalysis, and single-subject comparisons matched with longitudinal studies should be carried out. Second, sustainability issues should be carefully considered. Human, financial, and environmental resources should be targeted to investigate the affordability and suitability of such a proposed combination. Ethical issues should be additionally targeted. Third, a differentiation between the NDDs is currently lacking, with a large body of literature focused on ASDs. Other disorders, such as rare genetic diseases, should be included in future studies. Accordingly, future research perspectives should deal with the following topics: (a) empirical and/or experimental data to be collected, (b) systematic comparisons between groups or longitudinal investigations based on single subjects should be sought, (c) families' and caregivers' burden should be targeted, and (d) the clinical validity with a focus on sustainability and suitability in daily settings should be prioritized.
